# Complete genome sequence of *Cellulophaga lytica* type strain (LIM-21^T^)

**DOI:** 10.4056/sigs.1774329

**Published:** 2011-04-29

**Authors:** Amrita Pati, Birte Abt, Hazuki Teshima, Matt Nolan, Alla Lapidus, Susan Lucas, Nancy Hammon, Shweta Deshpande, Jan-Fang Cheng, Roxane Tapia, Cliff Han, Lynne Goodwin, Sam Pitluck, Konstantinos Liolios, Ioanna Pagani, Konstantinos Mavromatis, Galina Ovchinikova, Amy Chen, Krishna Palaniappan, Miriam Land, Loren Hauser, Cynthia D. Jeffries, John C. Detter, Evelyne-Marie Brambilla, K. Palani Kannan, Manfred Rohde, Stefan Spring, Markus Göker, Tanja Woyke, James Bristow, Jonathan A. Eisen, Victor Markowitz, Philip Hugenholtz, Nikos C. Kyrpides, Hans-Peter Klenk, Natalia Ivanova

**Affiliations:** 1 DOE Joint Genome Institute, Walnut Creek, California, USA; 2 DSMZ - German Collection of Microorganisms and Cell Cultures GmbH, Braunschweig, Germany; 3 Los Alamos National Laboratory, Bioscience Division, Los Alamos, New Mexico, USA; 4 Biological Data Management and Technology Center, Lawrence Berkeley National Laboratory, Berkeley, California, USA; 5 Oak Ridge National Laboratory, Oak Ridge, Tennessee, USA; 6 HZI – Helmholtz Centre for Infection Research, Braunschweig, Germany; 7 University of California Davis Genome Center, Davis, California, USA; 8 Australian Centre for Ecogenomics, School of Chemistry and Molecular Biosciences, The University of Queensland, Brisbane, Australia

**Keywords:** aerobic, motile by gliding, Gram-negative, agarolytic, chemoorganotrophic, *Flavobacteriaceae*, GEBA

## Abstract

*Cellulophaga lytica* (Lewin 1969) Johansen *et al.* 1999 is the type species of the genus *Cellulophaga,* which belongs to the family *Flavobacteriaceae* within the phylum '*Bacteroidetes*' and was isolated from marine beach mud in Limon, Costa Rica. The species is of biotechnological interest because its members produce a wide range of extracellular enzymes capable of degrading proteins and polysaccharides. After the genome sequence of *Cellulophaga algicola* this is the second completed genome sequence of a member of the genus *Cellulophaga*. The 3,765,936 bp long genome with its 3,303 protein-coding and 55 RNA genes consists of one circular chromosome and is a part of the *** G****enomic* *** E****ncyclopedia of* *** B****acteria and* *** A****rchaea * project.

## Introduction

Strain LIM-21^T^ (DSM 7489 = ATCC 23178 = JCM 8516) is the type strain of the species *Cellulophaga lytica*, which is the type species of the genus *Cellulophaga* [1]. The genus currently consists of five more validly named species [2]: *C. algicola* [3], *C*. *baltica, C*. *fucicola* [1], *C*. *pacifica* [4] and *C*. *tyrosinoxydans* [5]. The species was first described in 1969 by Lewin as '*Cytophaga lytica*' [6], and was subsequently transferred to the novel genus *Cellulophaga* as type strain *C. lytica* [1]. The genus name is derived from the Neo-Latin word 'cellulosum' meaning 'cellulose' and the latinized Greek word 'phagein' meaning 'to eat', yielding the Neo-Latin word 'Cellulophaga' meaning 'eater of cellulose' [2]. The species epithet is derived from the Neo-Latin word 'lytica' (loosening, dissolving) [2]. Here we present a summary classification and a set of features for *C. lytica* strain LIM-21^T^, together with the description of the complete genomic sequencing and annotation.

## Classification and features

A representative genomic 16S rRNA sequence of  strain LIM-21^T^ was compared using NCBI BLAST under default settings (e.g., considering only the high-scoring segment pairs (HSPs) from the best 250 hits) with the most recent release of the Greengenes database [7] and the relative frequencies, weighted by BLAST scores, of taxa and keywords (reduced to their stem [8]) were determined. The five most frequent genera were *Cellulophaga* (37.3%), *Flavobacterium* (8.5%), *Cytophaga* (6.3%), *Aquimarina* (5.8%) and *Arenibacter* (5.7%) (141 hits in total). Regarding the ten hits to sequences from members of the species, the average identity within HSPs was 99.0%, whereas the average coverage by HSPs was 93.3%. Regarding the eleven hits to sequences from other members of the genus, the average identity within HSPs was 94.0%, whereas the average coverage by HSPs was 93.1%. Among all other species, the one yielding the highest score was *Cytophaga lytica* (M62796), which corresponded to an identity of 99.2% and an HSP coverage of 96.9%. (Note that the Greengenes databases uses the INSDC (= EMBL/NCBI/DDBJ) annotation, which is not an authoritative source for nomenclature or classification).The highest-scoring environmental sequence was EU246790 ('Identification microorganism Libya untreated Mediterranean sea water feed reverse osmosis plant isolate RSW1-4RSW1-4 str. RSW1-4'), which showed an identity of 100.0% and an HSP coverage of 96.2%. The five most frequent keywords within the labels of environmental samples which yielded hits were 'sea' (5.6%), 'water' (4.8%), 'marin' (3.6%), 'sediment' (3.1%) and 'surfac' (2.8%) (109 hits in total). The single most frequent keyword within the labels of environmental samples which yielded hits of a higher score than the highest scoring species was 'feed, identif, libya, mediterranean, microorgan, osmosi, plant, revers, sea, untreat, water' (9.1%) (1 hit in total).

**Figure 1:**
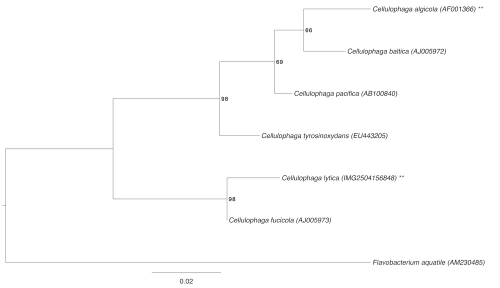
Phylogenetic tree highlighting the position of *C. lytica* relative to the other type strains within the genus. The tree was inferred from 1,458 aligned characters [9,10] of the 16S rRNA gene sequence under the maximum likelihood criterion [11] and rooted with the type strain of the type species of the family. The branches are scaled in terms of the expected number of substitutions per site. Numbers next to bifurcations are support values from 450 bootstrap replicates [12] if larger than 60%. Lineages with type strain genome sequencing projects that are registered in GOLD [13] but remain unpublished are labeled with one asterisk, published genomes with two asterisks [14].

Figure 1 shows the phylogenetic neighborhood of *C. lytica* in a 16S rRNA based tree. The sequence of the four 16S rRNA gene copies in the genome differ from each other by up to four nucleotides, and differ by up to 15 nucleotides from the previously published 16S rRNA sequence (D12666), which contains 19 ambiguous base calls.

**Figure 2:**
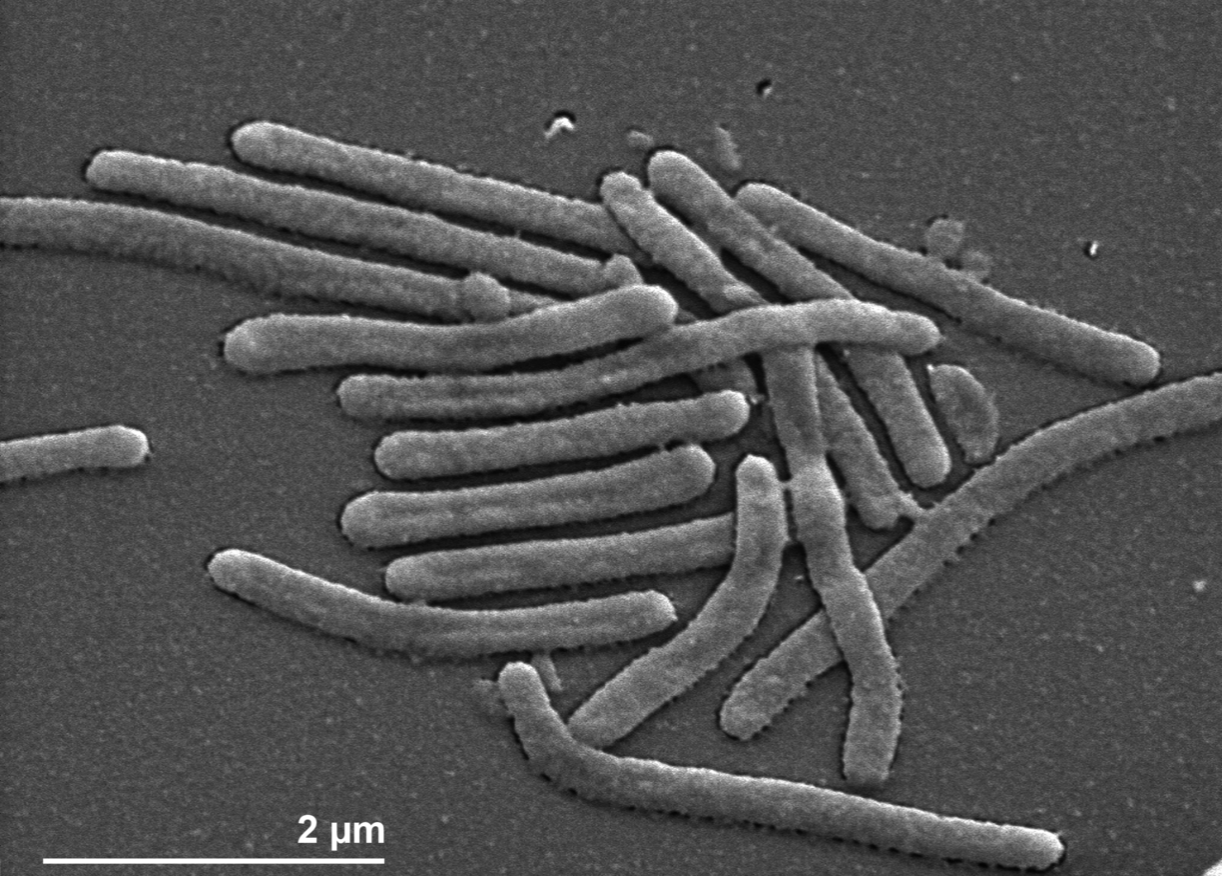
Scanning electron micrograph of *C*. *lytica* LIM-21^T^

The cells of *C*. *lytica* are slender flexible rods, cylindrical with blunt ends. Their lengths and widths range from 1.5-10 and 0.3-0.4 µm, respectively (Figure 2 and Table 1) [25]. *C*. *lytica* is motile by gliding [25]. Colonies have a bright yellow color caused by zeaxanthin as the main pigment; flexirubin-type pigments are not formed [24,28]. *C. lytica* requires Na^+^ and grows at NaCl concentrations up to 8% [3,5], in the presence of 10% NaCl no growth was observed [4]. The temperature range for growth is between 4°C [4] and 40°C [25], with an optimum between 22-30°C [25].

**Table 1:** Classification and general features of *C*. *lytica* LIM-21^T^ according to the MIGS recommendations [15].

MIGS ID	Property	Term	Evidence code
	Current classification	Domain *Bacteria*	TAS [16]
		Phylum '*Bacteroidetes*'	TAS [17]
		Class *Flavobacteria*	TAS [18]
		Order '*Flavobacteriales*'	TAS [19]
		Family *Flavobacteriaceae*	TAS [18,20-23]
		Genus *Cellulophaga*	TAS [1]
		Species *Cellulophaga lytica*	TAS [1]
		Type strain LIM-21	TAS [1]
	Gram stain	negative	TAS [24]
	Cell shape	rod-shaped	TAS [24]
	Motility	motile by gliding	TAS [24]
	Sporulation	none	TAS [24]
	Temperature range	4-40°C	TAS [4,25]
	Optimum temperature	22-30°C	TAS [25]
	Salinity	up to 8% NaCl	TAS [3,5]
MIGS-22	Oxygen requirement	aerobic	TAS [24]
	Carbon source	carbohydrates	TAS [24]
	Energy metabolism	chemoheterotroph	TAS [24]
MIGS-6	Habitat	mud	TAS [24]
MIGS-15	Biotic relationship	free-living	NAS
MIGS-14	Pathogenicity	none	NAS
	Biosafety level	1	TAS [26]
	Isolation	beach mud	TAS [24]
MIGS-4	Geographic location	Limon, Costa Rica	TAS [24]
MIGS-5	Sample collection time	1969	NAS
MIGS-4.1	Latitude	10.1	NAS
MIGS-4.2	Longitude	-83.5	NAS
MIGS-4.3	Depth	not reported	NAS
MIGS-4.4	Altitude	not reported	NAS

Evidence codes - IDA: Inferred from Direct Assay (first time in publication); TAS: Traceable Author Statement (i.e., a direct report exists in the literature); NAS: Non-traceable Author Statement (i.e., not directly observed for the living, isolated sample, but based on a generally accepted property for the species, or anecdotal evidence). These evidence codes are from of the Gene Ontology project [27]. If the evidence code is IDA, then the property was directly observed by one of the authors or an expert mentioned in the acknowledgements.

*C*. *lytica* is aerobic and chemoorganotrophic [24]. The organism can degrade agar, alginate, gelatin and starch [24,25], but not casein, cellulose (filter paper), chitin, alginic acid, elastin or fibrinogen [1,25]. There are conflicting observations describing the ability of *C. lytica* to degrade carboxymethylcellulose (CMC). Whereas most authors [3,5,24,25] describe the hydrolysis of CMC, Nedashkovskaya *et al*. 2004 [4] did not observe its degradation by *C. lytica.* Nitrate reduction and denitrification are negative [25]. *C. lytica* is catalase [24] and oxidase positive [25]. Acid is formed oxidatively from cellobiose, galactose, glucose, lactose, maltose and xylose [4]. *C*. *lytica* is sensitive to oleandomycin, lincomycin and shows resistance to benzylpenicillin, carbencillin, gentamicin, kanamycin, neomycin, ampicillin, streptomycin and tetracycline [4].

### Chemotaxonomy

The fatty acid profiles of four *C. lytica* strains were analyzed by Bowman in 2000 [3]. The predominant cellular acids of these four analyzed *C. lytica* strains were branched-chain saturated and unsaturated fatty acids and straight-chain saturated and monounsaturated fatty acids, namely i-C_15:0_ (18.9%), i-C_15:1_ω10c (10.3%), i-C_17:1_ω7c (5.1%), C_15:0_ (9.3%), C_16:1_ω7c (9.0%), i-C_15:0_ 3-OH (6.2%), i-C_16:0_ 3-OH (5.2%) and i-C_17:0_ 3-OH (20.8%) [3]. The isoprenoid quinones of *C*. *lytica* were not determined, but for *C*. *pacifica* the presence of MK-6 as the major lipoquinone was described [4]. Polar lipids have not been studied.

## Genome sequencing and annotation

### Genome project history

This organism was selected for sequencing on the basis of its phylogenetic position [29], and is part of the *** G****enomic* *** E****ncyclopedia of* *** B****acteria and* *** A****rchaea * project [30]. The genome project is deposited in the Genomes On Line Database [13] and the complete genome sequence is deposited in GenBank. Sequencing, finishing and annotation were performed by the DOE Joint Genome Institute (JGI). A summary of the project information is shown in Table 2.

**Table 2:** Genome sequencing project information

**MIGS ID**	**Property**	**Term**
MIGS-31	Finishing quality	Finished
MIGS-28	Libraries used	Three genomic libraries: one 454 pyrosequence standard library, one 454 PE library (8 kb insert size), one Illumina library
MIGS-29	Sequencing platforms	Illumina GAii, 454 GS FLX Titanium
MIGS-31.2	Sequencing coverage	1,605.2 × (Illumina); 22.9 × (pyrosequence)
MIGS-30	Assemblers	Newbler version 2.5-internal-10Apr08, Velvet version 0.7.63, phrap version SPS-4.24
MIGS-32	Gene calling method	Prodigal 1.4, GenePRIMP
	INSDC ID	CP002534
	Genbank Date of Release	February 28, 2011
	GOLD ID	Gc01668
	NCBI project ID	50743
	Database: IMG-GEBA	2504136007
MIGS-13	Source material identifier	DSM 7489
	Project relevance	Tree of Life, GEBA

### Growth conditions and DNA isolation

*C. lytica* LIM-21^T^, DSM 7489, was grown in DSMZ medium 514 (BACTO marine broth) [31] at 28°C. DNA was isolated from 0.5-1 g of cell paste using MasterPure Gram-positive DNA purification kit (Epicentre MGP04100) following the standard protocol as recommended by the manufacturer with modification st/DL for cell lysis as described in Wu *et al*. [30]. DNA is available through the DNA Bank Network [32].

### Genome sequencing and assembly

The genome was sequenced using a combination of Illumina and 454 sequencing platforms. All general aspects of library construction and sequencing can be found at the JGI website [33]. Pyrosequencing reads were assembled using the Newbler assembler version 2.5-internal-10Apr08 (Roche). The initial Newbler assembly consisting of 28 contigs in one scaffold was converted into a phrap version SPS - 4.24 [34] assembly by making fake reads from the consensus, to collect the read pairs in the 454 paired end library. Illumina GAii sequencing data (3,907 Mb) was assembled with Velvet [35] and the consensus sequences were shredded into 1.5 kb overlapped fake reads and assembled together with the 454 data. The 454 draft assembly was based on 156.1 Mb 454 draft data and all of the 454 paired end data. Newbler parameters are -consed -a 50 -l 350 -g -m -ml 20. The Phred/Phrap/Consed software package [34] was used for sequence assembly and quality assessment in the subsequent finishing process. After the shotgun stage, reads were assembled with parallel phrap (High Performance Software, LLC). Possible mis-assemblies were corrected with gapResolution [33], Dupfinisher [36], or sequencing cloned bridging PCR fragments with subcloning or transposon bombing (Epicentre Biotechnologies, Madison, WI). Gaps between contigs were closed by editing in Consed, by PCR and by Bubble PCR primer walks (J.-F.Chang, unpublished). A total of 238 additional reactions and two shatter libraries were necessary to close gaps and to raise the quality of the finished sequence. Illumina reads were also used to correct potential base errors and increase consensus quality using a software Polisher developed at JGI [37]. The error rate of the completed genome sequence is less than 1 in 100,000. Together, the combination of the Illumina and 454 sequencing platforms provided 1,628.1 × coverage of the genome. The final assembly contained 282,018 pyrosequence and 78,832,334 Illumina reads.

### Genome annotation

Genes were identified using Prodigal [38] as part of the Oak Ridge National Laboratory genome annotation pipeline, followed by a round of manual curation using the JGI GenePRIMP pipeline [39]. The predicted CDSs were translated and used to search the National Center for Biotechnology Information (NCBI) nonredundant database, UniProt, TIGR-Fam, Pfam, PRIAM, KEGG, COG, and InterPro databases. Additional gene prediction analysis and functional annotation was performed within the Integrated Microbial Genomes - Expert Review (IMG-ER) platform [40].

## Genome properties

**Figure 3:**
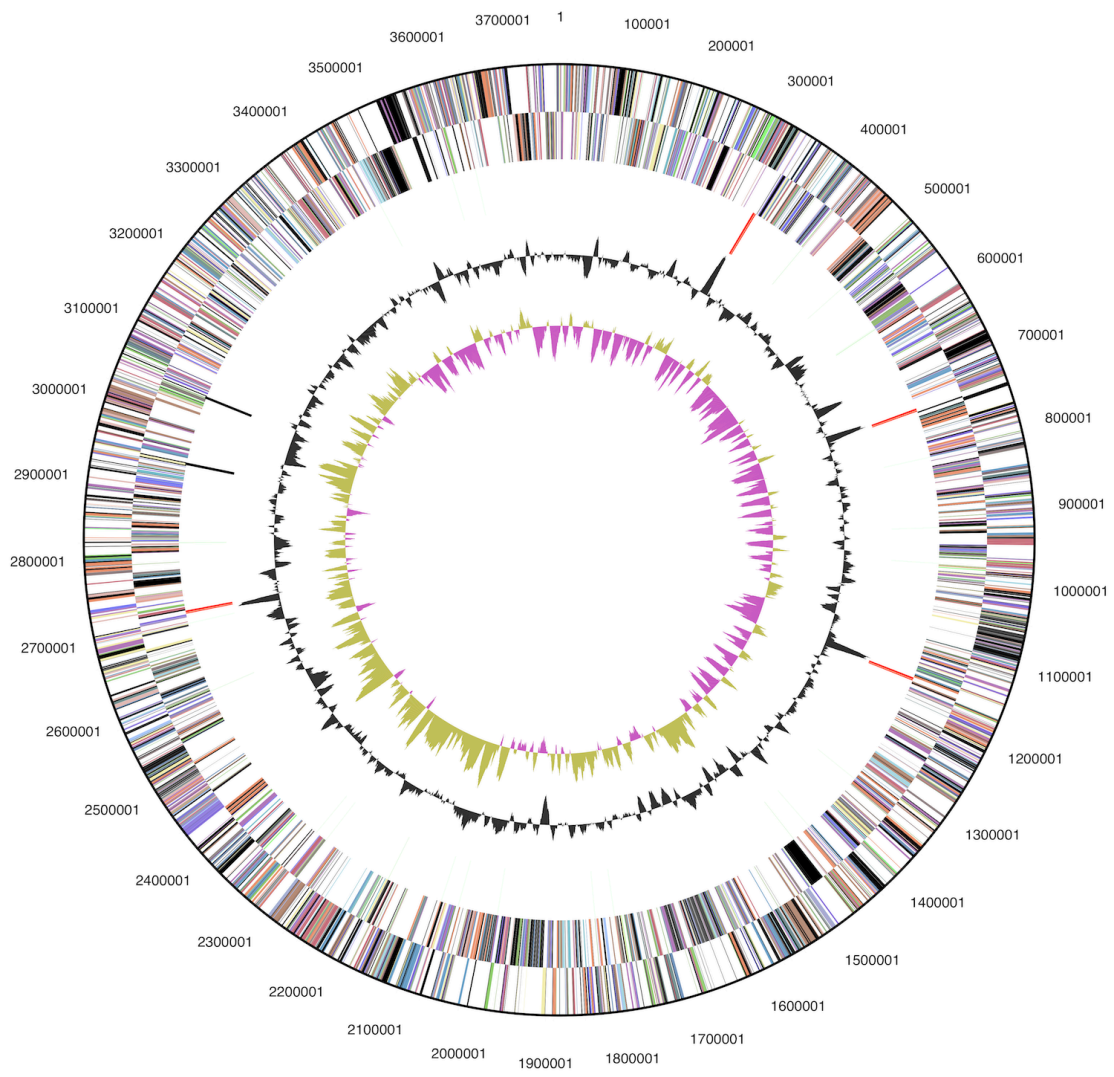
Graphical circular map of the chromosome. From outside to the center: Genes on forward strand (color by COG categories), Genes on reverse strand (color by COG categories), RNA genes (tRNAs green, rRNAs red, other RNAs black), GC content, GC skew.

The genome consists of a 3,765,936 bp long chromosome with a G+C content of 32.1% (Table 3 and Figure 3). Of the 3,358 genes predicted, 3,303 were protein-coding genes, and 55 RNAs; 19 pseudogenes were also identified. The majority of the protein-coding genes (65.5%) were assigned with a putative function while the remaining ones were annotated as hypothetical proteins. The distribution of genes into COGs functional categories is presented in Table 4.

**Table 3:** Genome Statistics

**Attribute**	**Value**	**% of Total**
Genome size (bp)	3,765,936	100.00%
DNA coding region (bp)	3,443,047	91.43%
DNA G+C content (bp)	1,209,276	32.11%
Number of replicons	1	
Extrachromosomal elements	0	
Total genes	3,358	100.00%
RNA genes	55	1.64%
rRNA operons	4	
Protein-coding genes	3,303	98.36%
Pseudo genes	19	0.57%
Genes with function prediction	2,200	65.52%
Genes in paralog clusters	344	10.24%
Genes assigned to COGs	2,098	62.48%
Genes assigned Pfam domains	2,346	69.86%
Genes with signal peptides	1,005	29.93%
Genes with transmembrane helices	794	23.65%
CRISPR repeats	0	

**Table 4:** Number of genes associated with the general COG functional categories

Code	value	%age	Description
J	154	6.7	Translation, ribosomal structure and biogenesis
A	0	0.0	RNA processing and modification
K	181	7.9	Transcription
L	107	4.7	Replication, recombination and repair
B	1	0.0	Chromatin structure and dynamics
D	18	0.8	Cell cycle control, cell division, chromosome partitioning
Y	0	0.0	Nuclear structure
V	41	1.8	Defense mechanisms
T	125	5.5	Signal transduction mechanisms
M	193	8.4	Cell wall/membrane/envelope biogenesis
N	4	0.2	Cell motility
Z	0	0.0	Cytoskeleton
W	0	0.0	Extracellular structures
U	30	1.3	Intracellular trafficking, secretion, and vesicular transport
O	99	4.3	Posttranslational modification, protein turnover, chaperones
C	122	5.3	Energy production and conversion
G	122	5.3	Carbohydrate transport and metabolism
E	194	8.5	Amino acid transport and metabolism
F	59	2.6	Nucleotide transport and metabolism
H	121	5.3	Coenzyme transport and metabolism
I	79	3.5	Lipid transport and metabolism
P	159	6.9	Inorganic ion transport and metabolism
Q	29	1.3	Secondary metabolites biosynthesis, transport and catabolism
R	276	12.1	General function prediction only
S	177	7.7	Function unknown
-	1,260	37.5	Not in COGs

## Insights from the genome sequence

A closer look at the genome sequence of strain LIM-21^T^ revealed a set of genes which might be responsible for the yellow-orange color of *C*. *lytica* cells by encoding enzymes that are involved in the synthesis of carotenoids. Carotenoids are produced by the action of geranylgeranyl pyrophosphate synthase (Celly_1682), phytoene synthase (Celly_0459), phytoene desaturase (Celly_0458), lycopene cyclase (Celly_0462) and carotene hydroxylase (Celly_0461). Geranylgeranyl pyrophosphate synthases start the biosynthesis of carotenoids by combining farnesyl pyrophosphate with C_5_ isoprenoid units to C_20_-molecules, geranylgeranyl pyrophosphate. The phytoene synthase catalyzes the condensation of two geranylgeranyl pyrophosphate molecules followed by the removal of diphosphate and a proton shift leading to the formation of phytoene. Sequential desaturation steps are conducted by the phytoene desaturase followed by cyclization of the ends of the molecules catalyzed by the lycopene cyclase [41]. This above mentioned set of genes was also found in the genome of *C. algicola* [14].

Strain LIM 21^T^ produces a wide range of extracellular enzymes degrading proteins and polysaccharides. *C*. *lytica*, like the other members of the genus *Cellulophaga*, cannot hydrolyze filter paper or cellulose in its crystalline form, though they can hydrolyze the soluble cellulose derivative carboxymethylcellulose (CMC). The genome sequence of strain LIM 21^T^ revealed the presence of three cellulases (Celly_0269, Celly_0304, Celly_0965), probably responsible for the hydrolysis of CMC. In addition two β-glucosidases (Celly_3249, Celly_1282) were identified in the genome, catalyzing the breakdown of the glycosidic β**-**1,4 bond between two glucose molecules in cellobiose. The deduced amino acid sequence of Celly_0304 shows 90% identity to the deduced sequence of the *C. algicola* cellulase coding gene Celly_0025. The identity of the deduced amino acid sequences of the cellulase encoding genes Celly_0269 and Celly_2753 is 65%. The neighborhoods of these two *C. lytica* cellulase genes have a similar structure like the respective genome regions in *C. algicola*, with orthologs belonging to different COG categories.

The LIM 21^T^ genome contains 15 genes coding for sulfatases, which are located in close proximity to glycoside hydrolase genes suggesting that sulfated polysaccharides may be used as substrates. α-L-fucoidan could be a substrate, as three α-L-fucosidases (Celly_0440, Celly_0442, Celly_0449) are located in close proximity to nine sulfatases (Celly_0432, Celly_0425, Celly_0426, Celly_0436, Celly_0431, Celly_0433, Celly_0435, Celly_0438, Celly_0444). Sakai and colleagues report the existence of intracellular α-L-fucosidases and sulfatases, which enable '*Fucophilus fucoidanolyticus*' to degrade fucoidan [42].

The above mentioned sulfatases and fucosidases containing region of *C. lytica* is similar to the recently described region of *C. algicola* with five α-L-fucosidases and three sulfatases [14]. This fucoidan degrading ability could be also shared by *Coraliomargarita akajimensis*, as the annotation of the genome sequence revealed the existence of 49 sulfatases and 12 α-L-fucosidases [43].

### Comparative genomics

The genomes of the two recently sequenced *Cellulophaga* type strains differ significantly in their size, *C. lytica* having 3.8 Mb and *C. algicola* 4.9 Mb and their number of pseudogenes, 19 (0.6%) and 122 (2.8%), respectively. Liu *et al*., 2004 have shown that pseudogenes in prokaryotes are not uncommon; the analysis of 64 genomes, including archaea, pathogen and nonpathogen bacteria, revealed an occurrence of pseudogenes of at least 1-5% of all gene-like sequences, with some genomes containing considerably higher amounts [44].

To estimate the overall similarity between the genomes of *C. lytica* and *C. algicola* the GGDC-Genome-to-Genome Distance Calculator [45,46] was used. The system calculates the distances by comparing the genomes to obtain HSPs (high-scoring segment pairs) and interfering distances from the set of formulas (1, HSP length / total length; 2, identities / HSP length; 3, identities / total length). The comparison of the genomes of *C. lytica* with *C. algicola* revealed that 25% of the average of both genome lengths are covered with HSPs. The identity within these HSPs was 82%, whereas the identity over the whole genome was only 20%. These results demonstrate that according to the whole genomes of *C. lytica* and *C. algicola* the similarity is not very high, although the comparison of 16S rRNA gene sequences showed only 7.7% differences.

In order to compare the *C. lytica* and *C. algicola* genomes, correlation values (Pearson coefficient) according to the similarity on the level of COG category, pfam, enzymes and TIGRfam were calculated. The highest correlation value (0.98) was reached on the level of pfam data; the correlation values on the basis of COG, enzyme and TIGRfam data were 0.88, 0.92 and 0.93, respectively. As a correlation value of 1 indicates the highest correlation, we can find a quite high correlation between the genomes of *C. lytica* and *C. algicola* at least considering the pfam data [40].

The comparison of the number of genes belonging to the different COG categories revealed no large differences in the genomes of *C. lytica* and *C. algicola*. A slightly higher fraction of genes belonging in the categories transcription (*C. lytica* 8.63%, *C. algicola* 6.85%), translation (*C. lytica* 7.34%, *C. algicola* 6.30%), amino acid metabolism (*C. lytica* 9.25%, *C. algicola* 8.19%), inorganic ion transport and metabolism (*C. lytica* 7.58%, *C. algicola* 6.85%) and posttranslational modification (*C. lytica* 4.72%, *C. algicola* 3.90%) were identified in *C. lytica*. The part of genes belonging to the following COG categories was slightly smaller in *C. lytica* than in *C. algicola*: carbohydrate metabolism (*C. lytica* 5.82%, *C. algicola* 6.77%), defense mechanisms (*C. lytica* 1.95%, *C. algicola* 2.48%), secondary metabolites biosynthesis (*C. lytica* 1.38%, *C. algicola* 2.05%).

**Figure 4:**
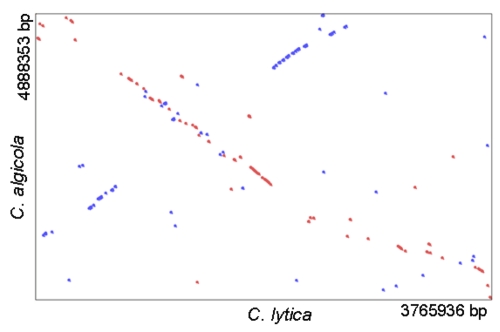
Synteny dot blot based on the genome sequences of *C. lytica* and *C. algicola*. Blue dots represent regions of similarity found on parallel strands and red dots show regions of similarity found on anti-parallel strands.

The synteny dot plot in Figure 4 shows a nucleotide based comparison of the genomes of *C. lytica* and *C. algicola*. Only in some parts of the genome a relatively high degree of similarity becomes visible. There exists a fragmented collinearity between the two genomes.

**Figure 5:**
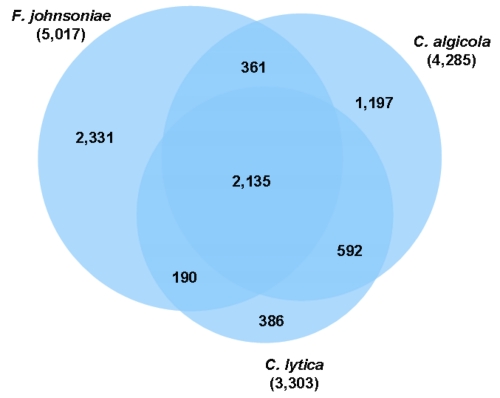
Venn-diagram depicting the intersections of protein sets (total numbers in parentheses) of *C. lytica*, *C. algicola* and *F. johnsoniae*.

The Venn-diagram (Figure 5) shows the number of shared genes. *C. lytica* and *C. algicola* share a great number of genes (592 genes) that are not present in the genome of *Flavobacterium johnsoniae* [47]. This fraction of genes includes genes coding for enzymes that are responsible for the degradation of polysaccharides, for example fucoidan and agar. While 15 sulfatases and three α-L-fucosidases were identified in the genome of *C. lytica,* and 22 sulfatases and five α-L-fucosidases were identified in the genome of *C. algicola*, only four sulfatase genes and no α-L-fucosidase genes were identified in the genome of *F. johnsoniae*. In addition, three agarases were identified in the genomes of *C. lytica* and *C. algicola*, each, whereas the genome of *F. johnsoniae* contains no agarase gene. *F. johnsoniae* is a chitin hydrolyzing organism; the genes involved in the utilization of chitin were described by McBride *et al.* (2009) [47]. *C. lytica* [1,25] and *C. algicola* [3] are non-chitinolytic, and there were no homologs to the chitin utilizing loci of *F. johnsoniae* identified in their genomes. To the group of genes that are shared by all three genomes belong the genes that code for enzymes which are involved in the biosynthesis of carotenoids, e.g. phytoene desaturase and phytoene synthase. But in contrast to the *Cellulophaga* species *F. johnsoniae* also produces flexirubin. The genes which are involved in the flexirubin synthesis of *F. johnsoniae* were identified by McBride *et al.* (2009) [47].
